# Remarks on Pulmonary Tuberculosis*Atlanta Society of Medicine, December 15, 1896.

**Published:** 1897-02

**Authors:** W. S. Kendrick

**Affiliations:** Atlanta, Ga.


### REMARKS ON PULMONARY TUBERCULOSIS.*

By W. S. KENDRICK, M.D.,
ATLANTA, GA.

I must offer an apology for having no paper this evening, but my work has lately been such as to render it impossible for me to prepare one. But I shall make some remarks upon the subject assigned me.

The trend of recent investigations goes to prove that tuberculosis is an infectious disease due to the presence of the tubercle bacillus. This attacks almost any portion of the body. The danger of infection is not from the moist state but from the expectorations of the persons having the disease. These expectorations become dry and are blown about in the air, and it is the inhalations of these that give rise to the most frequent forms of phthisis pulmonalis. We have several forms of this, but the form which I propose to discuss is the chronic ulcerative phthisis. That is the general form which we meet with in tuberculosis, and in which the process is slow, as the disease may run over a number of months or years, or may remain quiescent for a length of time. The inhaled bacilli usually attack the apex of the lung, and this is generally supposed to be due to the fact that the expansion here is less vigorous. Consequently the bacilli gain a vantage point there and set up the inflammation which we find accompanying tuberculosis. The disease is manifested by nodular bodies called tubercles, which we find in the bronchioles and elsewhere in the air passages. They excite a certain amount of inflammation wherever located, and this accounts for the fact that however small an area may be infected we never have a case without a case of secondary pneumonia. They are

*Atlanta Society of Medicine, December 15, 1896.

foreign bodies and give rise to secondary solidification of the lung tissue, as they excite a certain amount of inflammation, and whether as large as my finger or as small as a pin head we have the two forms of pneumonia. When the tubercles have existed for a certain length of time they die and produce softening and caseation; and in this breaking down of lung tissue the cavity is produced. We have two processes; one is caseation and the other is sclerosis. The life of the patient will depend upon which of these processes supervenes. In caseation we have softening and destruction of the lung tissue until we have the cavity, and this process repeats itself. The process of sclerosis is much more common than we usually think, and we would be surprised at the large number of persons over 45 years of age who have the sclerotic patches; forty or fifty per cent. of the people on whom post-mortems are made are found with these patches, and yet there was no evidence of tuberculosis during the patients' lives. As before stated, it is due to one or the other of these processes as to whether the patient will die early after the invasion or whether he will live longer.

Now as to the invasion of the tubercle. It is not suspected by the patient until he is notified. We have certain symptoms accompanying this; we sometimes have dyspepsia and derangement of the stomach, loss of flesh, loss of strength, emaciation, and a dry, hacking cough. One of the first signs is hemoptysis, or hemorrhage from the lungs. This is supposed to be nearly always due to the presence of the tubercles, which produce an erosion of some blood vessel and cause hemorrhage. Where we have a profuse hemorrhage it is usually due to the rupture of one of the large blood vessels in a cavity and results in the death of the patient. At first the cough is dry and hacking, for the reason that the destructive process has not yet become sufficiently advanced. The most characteristic sign is the fever. We may have a patient with night sweats and loss of flesh and yet not suspect phthisis, but, if, upon taking the temperature, we find the patient has a slight increase of temperature, particularly in the evening, which continues for weeks (ruling out other conditions) and he have the other symptoms, we can reach but one conclusion. The fever is pathognomonic and is the most reliable; if there is no typhoid fever or anything else, we can almost make out a perfect diagnosis, which will be confirmed by finding the bacilli.

We divide tuberculosis into three stages, whether acute or chronic. The first stage is the stage of acute pneumonia, that is, the stage of tuberculization; this is the irritative or congestive stage. The second stage corresponds to the second stage of acute pneumonia, that is, the stage of solidification. The third stage of acute pneumonia is the stage of resolution, and it is at this point that these two diseases separate. In acute croupous pneumonia we have resolution and the walls return to the normal condition. In ordinary croupous pneumonia we have no destruction of lung tissue. The two diseases diverge at this point, for instead of tuberculosis terminating in resolution we have the other process which is directly opposite, and we have caseation, softening and destruction of lung tissue, however small or large the affected area may be, and thereby giving rise to cavities.

Now we come to the physical signs. In the incipient stage the physical signs are absent to such an extent that we cannot say whether it is tuberculosis or not. After the disease has reached a certain point, we value inspection, percussion, palpation and auscultation; especially auscultation and percussion, and percussion is the more valuable of the two. Inspection gives us very little; if we get anything at all it is a certain amount of lost expansion at the apex, but in many cases we will not get even this. Of course, if the disease is well advanced we get on palpation an increase of vocal fremitus. This is typical: if we lay our hands on both lungs and ask the patient to speak we perceive the difference in the fremitus on the two sides. If, in the first or second stage we get great loss of expansion, we know exactly what is going to follow; we know that we will get an increase of vocal fremitus and have dullness upon percussion, and upon auscultation we will get broncho-vesicular breathing. We will get harsh inspiration and prolonged expiration. If we listen at the beginning of the invasion of the disease we will nearly always get prolonged expiration. We get this because the alveoli are congested; the lungs lose their elasticity and it takes the air longer to escape.

The second and third stages run so close together that one description will answer for both, except when we have cavities. In the second stage of tuberculosis, which is the pneumonic stage, we get loss of expansion. I will say just here that in making examinations, it is impossible to do this thoroughly if the patients are allowed to retain any of their clothes around the thorax; they should be stripped to the waist. The loss of expansion is due to the inflammatory changes in the lungs and also due to pleurisy. We know that we will get dullness on percussion after loss of expansion, and then when we listen we get the strongest sign, which is bronchial breathing. What we hear we get on expiration, because the inspiration is active and the expiration passive. In normal conditions the pitch of inspiration is higher and of expiration lower, and the reverse is true in all conditions of solidified lungs. In solidified lungs the pitch is higher and longer in expiration and gets higher until respiration ceases.

In the third stage we get practically the same signs; we get softening of lung tissue and cavities. All three stages are going on at the same time. The third stage differs from the second upon auscultation, as we have various sounds in the third stage. These sounds are indicative of cavities filled with air or filled with fluid, or partly filled with air and partly filled with fluid. These are some of the physical signs. Of course, I have left out a great deal of it, as it is something that one could talk on for hours, but these are what we find in ordinary cases of tuberculosis. One thing especially, we do not find all these signs on the anterior surface of the chest, and we hardly get them at the apex, but usually below the clavicle, not at the apex itself, but a little lower down. We may get well marked solidification and cavities on the posterior portion before any signs appear on the anterior surface of the chest.

As regards prognosis we cannot say in all these cases that death will be the result, for many cases are retrogressive and the patient will live for a number of years and go on discharging his duties. Death, of course, comes to a number of cases from many causes. In the latter stages the disease becomes a septic disease in which the tubercle may not play an important part, but a general septic condition is produced and death is due to sepsis.

